# Supplementary material for: A survey of Cryptosporidium prevalence among birds in two zoos in China
Source: PeerJ. 2022 Jan 19;10:e12825. doi: 10.7717/peerj.12825 (PMC8783555; doi:10.7717/peerj.12825)
Supplement: Supplemental Information 1 [file peerj-10-12825-s001.docx]

Additional file 1

Table S1 The details of birds in Zoo involved in the present research

| Host species | Genus | Family | Fecal sample numbers | Origin |
| --- | --- | --- | --- | --- |
| *Cygnus cygnus* | *Cygnus* | Anatidae | 4 | BJZ |
| *Cygnus atratus* | *Cygnus* | Anatidae | 8 | BJZ |
| *Cygnus* | *Cygnus* | Anatidae | 1 | BJZ |
| *Anser indicus* | *Anser* | Anatidae | 7 | BJZ（6） |
|  |  |  |  | HNFZ（1） |
| *Anser cygnoides* | *Anser* | Anatidae | 1 | HNFZ |
| *Anas platyrhynchos* | *Anas* | Anatidae | 4 | BJZ（2） |
|  |  |  |  | HNFZ（2） |
| *Tadorna ferruginea* | *Anas* | Anatidae | 3 | BJZ |
| *Branta canadensis* | *Branta* | Anatidae | 2 | BJZ |
| *Aythya nyroca* | *Aythya* | Anatidae | 2 | BJZ |
| *Nycticorax nycticorax* | *Nycticorax* | Ardeidae | 1 | BJZ |
| *Anthracoceros albirostris* | *Buceros* | Bucerotidae | 3 | HNFZ |
| *Leptoptilos javanicus* | *Leptoptilos* | Ciconiidae | 4 | BJZ |
| *Goura scheepmakeri* | *Ocyphapslophotes* | Columbidae | 1 | BJZ |
| *Columba* | *Columba* | Columbidae | 3 | HNFZ |
| *Crax alector* | *Crax* | Cracidae | 1 | BJZ |
| *Lonchura malacca* | *Lonchura* | Estrildidae | 1 | BJZ |
| *Grus monacha* | *Grus* | Gruidae | 4 | BJZ |
| *Grus japonensis* | *Grus* | Gruidae | 24 | BJZ（12） |
|  |  |  |  | HNFZ（12） |
| *Grus leucogeranus* | *Grus* | Gruidae | 35 | BJZ（34） |
|  |  |  |  | HNFZ（1） |
| *Grus vipio* | *Grus* | Gruidae | 15 | BJZ（9） |
|  |  |  |  | HNFZ（6） |
| *Grus nigricollis* | *Grus* | Gruidae | 11 | BJZ |
| *Balearica regulorum* | *Balearica* | Gruidae | 25 | BJZ（1） |
|  |  |  |  | HNFZ（24） |
| *Anthropoides virgo* | *Anthropoides* | Gruidae | 4 | HNFZ |
| *Numididae* | *Agelastes* | Numididae | 3 | HNFZ |
| *Eophona migratoria* | *Eophona* | Passeridae | 2 | BJZ |
| *Tetraogallus* | *Tetraogallus* | Phasianidae | 1 | BJZ |
| *Tetraogallus himalayensis* | *Tetraogallus* | Phasianidae | 1 | BJZ |
| *Crossoptilon crossoptilon* | *Crossoptilon* | Phasianidae | 1 | BJZ |
| *Crossoptilon mantchuricum* | *Crossoptilon* | Phasianidae | 1 | BJZ |
| *Crossoptilon harmani* | *Crossoptilon* | Phasianidae | 1 | BJZ |
| *Crossoptilon auritum* | *Crossoptilon* | Phasianidae | 1 | BJZ |
| *Syrmaticus reevesii* | *Syrmaticus* | Phasianidae | 1 | BJZ |
| *Phasianus colchicus* | *Phasianus* | Phasianidae | 1 | BJZ |
| *Lophura nycthemera* | *Lophura* | Phasianidae | 2 | BJZ |
| *Lophura swinhoii* | *Lophura* | Phasianidae | 1 | BJZ |
| *Chrvsolophus amherstiae* | *Chrysolophus* | Phasianidae | 3 | BJZ |
| *Tragopan caboti* | *Tragopan* | Phasianidae | 1 | BJZ |
| *Pavonini* | *Pavo* | Phasianidae | 4 | HNFZ |
| *Lonchura malacca* | *Lonchura* | Ploceidae | 1 | BJZ |
| *Diopsittaca nobilis* | *Diopsittaca nobilis* | Psittacidae | 1 | BJZ |
| *Phoenicopteridae* | *Phoeniconaias* | Phoenicopteridae | 68 | BJZ (66) |
|  |  |  |  | HNFZ (2) |
| *Mycteria ibis* | *Mycteria* | Threskiorothidae | 4 | BJZ |
| *laughing thrush* | *Garrulax* | Timaliidae | 1 | BJZ |
| *Total* | *-* | - | 263 | BJZ (197)  HNFZ (66) |

Abbreviations: BJZ, Beijing Zoo; HNFZ, Harbin Northern Forest Zoo
