# Supplementary material for: A survey of Cryptosporidium prevalence among birds in two zoos in China
Source: PeerJ. 2022 Jan 19;10:e12825. doi: 10.7717/peerj.12825 (PMC8783555; doi:10.7717/peerj.12825)
Supplement: Supplemental Information 2 [file peerj-10-12825-s002.docx]

Table S2 The haplotype of 8 different *Cryptosporidium* species downloaded from GenBank database

| Species | GenBank | Host | Country | Haplotype |
| --- | --- | --- | --- | --- |
| *C.parvum* | MF462154.1 | *Psittacula krameri* | Brazil | Hap1 |
|  | KY514066.1 | *Columba livia* | Brazil | Hap1 |
|  | MF400849.1 | *Anas platyrhynchos* | Nigeria | Hap2 |
|  | MF400845.1 | *Anas platyrhynchos* | Nigeria | Hap2 |
|  | MF462153.1 | *Forpus* sp. | Brazil | Hap3 |
|  | MG209077.1 | *Gallus gallus* | Brazil | Hap3 |
|  | KY514062.1 | *Columba livia* | Brazil | Hap3 |
|  | KT151554.1 | *Gallus gallus* | Iraq | Hap3 |
|  | KT151548.1 | *Coturnix coturnix* | Iraq | Hap3 |
|  | KT151547.1 | *Columba livia* | Iraq | Hap3 |
|  | KT151540.1 | *Columba livia* | Iraq | Hap3 |
|  | KT151536.1 | *Anas platyrhynchos* | Iraq | Hap3 |
|  | KT151524.1 | *Anas platyrhynchos* | Iraq | Hap3 |
|  | KT151529.1 | *Meleagris gallopavo* | Iraq | Hap4 |
| *C.parvum* in present study | MW664001.1 | *Grus leucogeranus* | China | Hap1 |
|  | MW664002.1 | *Grus leucogeranus* | China | Hap1 |
|  | MW664003.1 | *Grus leucogeranus* | China | Hap1 |
|  | MW664005.2 | *Phoenicopteridae* | China | Hap1 |
|  | MW664006.1 | *Grus leucogeranus* | China | Hap5 |
| *C.meleagridis* | AF112574.1 | *Meleagris gallopavo* | America | Hap1 |
|  | EU827314.1 | *Gallus gallus domesticus* | China | Hap1 |
|  | AJ493207.1 | *Meleagris gallopavo* | Thailand | Hap1 |
|  | AJ493549.1 | *Meleagris gallopavo* | Kenya | Hap1 |
|  | MH062745.1 | *Coturnix coturnix* | Brazil | Hap1 |
|  | MH062744.1 | *Gallus gallus domesticus* | Brazil | Hap1 |
|  | MH062743.1 | *Meleagris gallopavo* | Brazil | Hap1 |
|  | MG516759.1 | *Aves* | Australia | Hap1 |
|  | MG209078.1 | *Gallus gallus* | Brazil | Hap1 |
|  | JX548300.1 | *Gallus gallus domesticus* | China | Hap1 |
|  | JQ217141.1 | *Coturnix coturnix* | China | Hap1 |
|  | HM002494.1 | *Gallus gallus* | China | Hap1 |
|  | EU814439.1 | *Columba* | China | Hap1 |
|  | HM116384.1 | *Streptopelia orientalis* | China | Hap1 |
|  | HM116382.1 | *Columba livia* | China | Hap1 |
|  | EU717830.1 | *Coturnix coturnix* | China | Hap1 |
|  | AF180339.1 | *Psittacula krameri* | India | Hap1 |
|  | KT151551.1 | *Gallus gallus* | Iraq | Hap1 |
|  | KT151539.1 | *Meleagris gallopavo* | Iraq | Hap1 |
|  | AJ493208.1 | *Meleagris gallopavo* | Thailand | Hap2 |
|  | KT151549.1 | *Columba livia* | Iraq | Hap3 |
| *C.avium* | KU058875.1 | *Cygnus cygnus* | China | Hap1 |
| *C.baileyi* | AY954884.1 | *Gallus gallus domesticus* | China | Hap1 |
|  | EU827310.1 | *Gallus gallus domesticus* | China | Hap1 |
|  | EU741836.1 | *Struthio camelus* | China | Hap1 |
|  | EU741831.1 | *Struthio camelus* | China | Hap1 |
|  | AY954883.1 | *Gallus gallus domesticus* | China | Hap1 |
|  | MH062742.1 | *Coturnix coturnix* | Brazil | Hap1 |
|  | MH062741.1 | *Gallus gallus domesticus* | Brazil | Hap1 |
|  | MF627420.1 | *Gallus gallus domesticus* | Brazil | Hap1 |
|  | MG209080.1 | *Gallus gallus* | Brazil | Hap1 |
|  | MG209079.1 | *Gallus gallus* | Brazil | Hap1 |
|  | KY448455.1 | *Gallus gallus domesticus* | China | Hap1 |
|  | JQ217142.1 | *Coturnix coturnix* | China | Hap1 |
|  | HM002495.1 | *Gallus gallus* | China | Hap1 |
|  | EU814431.1 | *Anatinae* | China | Hap1 |
|  | HM116380.1 | *Pica hudsonia* | China | Hap1 |
|  | HM116378.1 | *Taeniopygia guttata* | China | Hap1 |
|  | HM116379.1 | *Galerida cristata* | China | Hap1 |
|  | HM116377.1 | *Chloebia gouldiae* | China | Hap1 |
|  | HM116376.1 | *Padda oryzivora* | China | Hap1 |
|  | HM116375.1 | *Leiothrix lutea* | China | Hap1 |
|  | HM116374.1 | *Acridotheres tristis* | China | Hap1 |
|  | GU377270.1 | *Struthio camelus* | China | Hap1 |
|  | GU377276.1 | *Struthio camelus* | China | Hap1 |
|  | EU717828.1 | *Coturnix coturnix* | China | Hap1 |
|  | DQ898161.1 | *Coturnix coturnix* | China | Hap1 |
|  | KP198614.1 | *Gallus gallus domesticus* | China | Hap1 |
|  | KM977661.1 | *Grus japonensis* | China | Hap1 |
|  | KM581271.1 | *Anatinae* | Egypt | Hap1 |
|  | KT151542.1 | *Columba livia* | Iraq | Hap1 |
|  | KT151544.1 | *Gallus gallus* | Iraq | Hap1 |
|  | KT151550.1 | *Coturnix coturnix* | Iraq | Hap1 |
|  | KX962159.1 | *Lonchura* sp. | China | Hap1 |
|  | AY954882.1 | *Anatinae* | China | Hap2 |
|  | KU744847.1 | *Gallus domesticlus brisson* | China | Hap2 |
|  | KU744845.1 | *Acridotheres cristatellus* | China | Hap3 |
|  | KU744846.1 | *Serinus canaria* | China | Hap4 |
|  | JX548297.1 | *Gallus gallus domesticus* | China | Hap5 |
|  | DQ060421.1 | *Gallus gallus domesticus* | China | Hap6 |
| *C.muris* | GQ227706.1 | *Struthio camelus* | China | Hap1 |
| *C.andersoni* | MN379937.1 | *Cygnus cygnus* | China | Hap1 |
|  | MN379938.1 | *Cygnus cygnus* | China | Hap1 |
|  | MN379941.1 | *Cygnus cygnus* | China | Hap1 |
|  | MN379940.1 | *Cygnus cygnus* | China | Hap2 |
|  | MN379942.1 | *Cygnus cygnus* | China | Hap3 |
| *C.galli* | MF405452.1 | *Zenaida auriculata* | Brazil | Hap1 |
|  | MF405449.1 | *Zenaida auriculata* | Brazil | Hap2 |
|  | MG516766.1 | *Threskiornithinae* | Australia | Hap3 |
|  | KU744848.1 | *Garrulax canorus* | China | Hap4 |
|  | KU221106.1 | *Phasianus colchicus* | Czech Republic | Hap5 |
|  | GU734645.1 | *Oryzoborus maximiliani* | Brazil | Hap5 |
|  | EU543269.1 | *Serinus canaria* | Brazil | Hap5 |
|  | HM116388.1 | *Bombycilla garrulus* | China | Hap6 |
|  | HM116387.1 | *Leiothrix argentauris* | China | Hap6 |
|  | KT151543.1 | *Coturnix coturnix* | Iraq | Hap6 |
|  | KT151553.1 | *Gallus gallus* | Iraq | Hap6 |
|  | GU734647.1 | *Cyanocompsa brissonii* | Brazil | Hap7 |
|  | GU734646.1 | *Sporophila collaris* | Brazil | Hap7 |
|  | EU543270.1 | *Oryzoborus angolensis* | Brazil | Hap8 |
|  | EU543268.1 | *Nymphicus hollandicus* | Brazil | Hap8 |
