# Supplementary material for: A survey of Cryptosporidium prevalence among birds in two zoos in China
Source: PeerJ. 2022 Jan 19;10:e12825. doi: 10.7717/peerj.12825 (PMC8783555; doi:10.7717/peerj.12825)
Supplement: Supplemental Information 3 [file peerj-10-12825-s003.docx]

Table S3 The haplotypes of *C.parvum* of 4 different hosts classes

| Host | Haplotypes | GenBank | Country | Haplotype |
| --- | --- | --- | --- | --- |
| Cattle | Hap1-7 | AB513857.1 | Egypt | Hap1 |
|  |  | AB513858.1 | Egypt | Hap1 |
|  |  | AB513859.1 | Egypt | Hap1 |
|  |  | AB513860.1 | Egypt | Hap1 |
|  |  | AB513861.1 | Egypt | Hap1 |
|  |  | AB513862.1 | Egypt | Hap1 |
|  |  | AB513863.1 | Egypt | Hap1 |
|  |  | AB513864.1 | Egypt | Hap1 |
|  |  | AB513865.1 | Egypt | Hap1 |
|  |  | AB513866.1 | Egypt | Hap1 |
|  |  | AB513867.1 | Egypt | Hap1 |
|  |  | AB513868.1 | Egypt | Hap1 |
|  |  | AB513870.1 | Egypt | Hap1 |
|  |  | AB513871.1 | Egypt | Hap1 |
|  |  | AB513872.1 | Egypt | Hap1 |
|  |  | AB513873.1 | Egypt | Hap1 |
|  |  | AB513874.1 | Egypt | Hap1 |
|  |  | AB513875.1 | Egypt | Hap1 |
|  |  | AB513876.1 | Egypt | Hap1 |
|  |  | AB513877.1 | Egypt | Hap1 |
|  |  | AB513878.1 | Egypt | Hap1 |
|  |  | AB513879.1 | Egypt | Hap1 |
|  |  | AB513880.1 | Egypt | Hap1 |
|  |  | AB513881.1 | Egypt | Hap1 |
|  |  | AB746195.1 | Japan | Hap1 |
|  |  | AB777179.1 | Egypt | Hap1 |
|  |  | AB777180.1 | Egypt | Hap1 |
|  |  | AB777181.1 | Egypt | Hap1 |
|  |  | AB777182.1 | Egypt | Hap1 |
|  |  | AB777183.1 | Egypt | Hap1 |
|  |  | AB777184.1 | Egypt | Hap1 |
|  |  | AB777185.1 | Egypt | Hap1 |
|  |  | AB777186.1 | Egypt | Hap1 |
|  |  | AB777187.1 | Egypt | Hap1 |
|  |  | AB777188.1 | Egypt | Hap1 |
|  |  | AB909499.1 | Japan | Hap1 |
|  |  | AB922117.1 | Egypt | Hap1 |
|  |  | AB922118.1 | Egypt | Hap1 |
|  |  | AB968047.1 | Japan | Hap1 |
|  |  | AB968048.1 | Japan | Hap1 |
|  |  | AJ493075.1 | Britain | Hap1 |
|  |  | AJ493090.1 | Kenya | Hap1 |
|  |  | AJ493201.1 | Thailand | Hap1 |
|  |  | AY204237.1 | America | Hap1 |
|  |  | AY204238.1 | America | Hap1 |
|  |  | KX266232.1 | China | Hap1 |
|  |  | KX929930.1 | Brazil | Hap1 |
|  |  | KX929931.1 | Brazil | Hap1 |
|  |  | KX929932.1 | Brazil | Hap1 |
|  |  | KX929933.1 | Brazil | Hap1 |
|  |  | KX929934.1 | Brazil | Hap1 |
|  |  | KX929935.1 | Brazil | Hap1 |
|  |  | KX929936.1 | Brazil | Hap1 |
|  |  | KX929937.1 | Brazil | Hap1 |
|  |  | KX929938.1 | Brazil | Hap1 |
|  |  | KX929939.1 | Brazil | Hap1 |
|  |  | KX929940.1 | Brazil | Hap1 |
|  |  | KX929941.1 | Brazil | Hap1 |
|  |  | KX929942.1 | Brazil | Hap1 |
|  |  | KX929943.1 | Brazil | Hap1 |
|  |  | KX929944.1 | Brazil | Hap1 |
|  |  | KX929945.1 | Brazil | Hap1 |
|  |  | KX929946.1 | Brazil | Hap1 |
|  |  | KX929947.1 | Brazil | Hap1 |
|  |  | KX929948.1 | Brazil | Hap1 |
|  |  | KX929949.1 | Brazil | Hap1 |
|  |  | KX929950.1 | Brazil | Hap1 |
|  |  | KX929951.1 | Brazil | Hap1 |
|  |  | KX929952.1 | Brazil | Hap1 |
|  |  | KX929953.1 | Brazil | Hap1 |
|  |  | KX929954.1 | Brazil | Hap1 |
|  |  | KX929956.1 | Brazil | Hap1 |
|  |  | LC012015.1 | Japan | Hap1 |
|  |  | LC012016.1 | Japan | Hap1 |
|  |  | JQ250803.1 | Turkey | Hap2 |
|  |  | JQ250804.1 | Turkey | Hap3 |
|  |  | LC620456.1 | India | Hap4 |
|  |  | AJ493085.1 | Kenya | Hap5 |
|  |  | AJ493071.1 | Kenya | Hap6 |
|  |  | FJ039877.1 | Saudi Arabia | Hap7 |
| Dog | Hap1,5,8-10 | MF589922.1 | Brazil | Hap1 |
|  |  | MF589923.1 | Brazil | Hap5 |
|  |  | KX811734.1 | Nigeria | Hap8 |
|  |  | GQ865532.1 | Chile | Hap9 |
|  |  | GQ865530.1 | Chile | Hap10 |
| Human | Hap1,5,11-17 | AB830575.1 | Ethiopia | Hap1 |
|  |  | AB830576.1 | Ethiopia | Hap1 |
|  |  | AB830578.1 | Ethiopia | Hap1 |
|  |  | AB830580.1 | Ethiopia | Hap1 |
|  |  | AB830581.1 | Ethiopia | Hap1 |
|  |  | AB830582.1 | Ethiopia | Hap1 |
|  |  | AJ849461.1 | Slovenia | Hap1 |
|  |  | AY204236.1 | Britain | Hap1 |
|  |  | DQ182559.1 | Norway | Hap1 |
|  |  | DQ388387.1 | Netherlands | Hap1 |
|  |  | GQ368454.1 | Chile | Hap1 |
|  |  | JX237833.1 | Egypt | Hap1 |
|  |  | JX298596.1 | Egypt | Hap1 |
|  |  | JX298597.1 | Egypt | Hap1 |
|  |  | JX298598.1 | Egypt | Hap1 |
|  |  | JX298600.1 | Egypt | Hap1 |
|  |  | KF928957.1 | Spain | Hap1 |
|  |  | KM012040.1 | / | Hap1 |
|  |  | KM012042.1 | / | Hap1 |
|  |  | KM012043.1 | / | Hap1 |
|  |  | KM012044.1 | / | Hap1 |
|  |  | KM012045.1 | / | Hap1 |
|  |  | KM012046.1 | / | Hap1 |
|  |  | KM085018.1 | / | Hap1 |
|  |  | KM215739.1 | Lebanon | Hap1 |
|  |  | KM215740.1 | Lebanon | Hap1 |
|  |  | KM215741.1 | Lebanon | Hap1 |
|  |  | KM215742.1 | Lebanon | Hap1 |
|  |  | KM215743.1 | Lebanon | Hap1 |
|  |  | KM285241.1 | Iran | Hap1 |
|  |  | KM285251.1 | Iran | Hap1 |
|  |  | KM285252.1 | Iran | Hap1 |
|  |  | KM285257.1 | Iran | Hap1 |
|  |  | KP204486.1 | Iraq | Hap1 |
|  |  | KP213126.1 | Iraq | Hap1 |
|  |  | KX216598.1 | Iran | Hap1 |
|  |  | KX685191.1 | Iran | Hap1 |
|  |  | MG969537.1 | Iran | Hap1 |
|  |  | AB830579.1 | Ethiopia | Hap5 |
|  |  | AB830583.1 | Ethiopia | Hap5 |
|  |  | AB830584.1 | Ethiopia | Hap5 |
|  |  | AJ849463.1 | Slovenia | Hap5 |
|  |  | DQ388388.1 | Netherlands | Hap5 |
|  |  | KM285245.1 | Iran | Hap5 |
|  |  | KM285246.1 | Iran | Hap5 |
|  |  | KM285247.1 | Iran | Hap5 |
|  |  | KM285248.1 | Iran | Hap5 |
|  |  | AY204227.1 | Britain | Hap11 |
|  |  | AJ493197.1 | Thailand | Hap12 |
|  |  | AJ493527.1 | Kenya | Hap13 |
|  |  | AJ493074.1 | Kenya | Hap14 |
|  |  | KM285242.1 | Iran | Hap15 |
|  |  | KM285243.1 | Iran | Hap15 |
|  |  | KM285244.1 | Iran | Hap15 |
|  |  | KM285253.1 | Iran | Hap16 |
|  |  | KM285254.1 | Iran | Hap16 |
|  |  | KM285255.1 | Iran | Hap16 |
|  |  | KM285256.1 | Iran | Hap16 |
|  |  | KP213123.1 | Iraq | Hap17 |
|  |  | KP213124.1 | Iraq | Hap17 |
|  |  | KP213125.1 | Iraq | Hap17 |
| Birds | Hap1,4,5 | FJ984565.1 | Hungary | Hap1 |
|  |  | HM059831.1 | / | Hap1 |
|  |  | KT151524.1 | Iraq | Hap1 |
|  |  | KT151529.1 | Iraq | Hap1 |
|  |  | KT151536.1 | Iraq | Hap1 |
|  |  | KT151540.1 | Iraq | Hap1 |
|  |  | KT151547.1 | Iraq | Hap1 |
|  |  | KT151548.1 | Iraq | Hap1 |
|  |  | KT151552.1 | Iraq | Hap1 |
|  |  | KT151554.1 | Iraq | Hap1 |
|  |  | KY514062.1 | Brazil | Hap1 |
|  |  | MF462153.1 | Brazil | Hap1 |
|  |  | MW664006.1 G4 | China | Hap4 |
|  |  | KY514066.1 | Brazil | Hap5 |
|  |  | MF462154.1 | Brazil | Hap5 |
|  |  | MF627421.1 | Brazil | Hap5 |
|  |  | MW664001.1 G3 | China | Hap5 |
|  |  | MW664002.1 G5 | China | Hap5 |
|  |  | MW664003.1 G6 | China | Hap5 |
|  |  | MW664005.2 P1 | China | Hap5 |
