# Supplementary material for: A survey of Cryptosporidium prevalence among birds in two zoos in China
Source: PeerJ. 2022 Jan 19;10:e12825. doi: 10.7717/peerj.12825 (PMC8783555; doi:10.7717/peerj.12825)
Supplement: Supplemental Information 4 [file peerj-10-12825-s004.docx]

Table S4 The details of *C.parvum* of 3 countries

| Country | GenBank | Host |
| --- | --- | --- |
| Brazil | MF462154.1 | *Psittacula krameri* |
| Brazil | MF462153.1 | *Forpus* sp. |
| Brazil | MG209077.1 | *Gallus gallus* |
| Brazil | KY514066.1 | *Columba livia* |
| Brazil | KY514065.1 | *Columba livia* |
| Brazil | KY514064.1 | *Columba livia* |
| Brazil | KY514063.1 | *Columba livia* |
| Brazil | KY514062.1 | *Columba livia* |
| Iraq | KT151554.1 | *Gallus gallus* |
| Iraq | KT151552.1 | *Gallus gallus* |
| Iraq | KT151548.1 | *Coturnix coturnix* |
| Iraq | KT151547.1 | *Columba livia* |
| Iraq | KT151540.1 | *Columba livia* |
| Iraq | KT151536.1 | *Anas platyrhynchos* |
| Iraq | KT151529.1 | *Meleagris gallopavo* |
| Iraq | KT151524.1 | *Anas platyrhynchos* |
| China | MW664001.1 | *Grus leucogeranus* |
| China | MW664002.1 | *Grus leucogeranus* |
| China | MW664003.1 | *Grus leucogeranus* |
| China | MW664005.2 | *Phoenicopteridae* |
| China | MW664006.1 | *Grus leucogeranus* |
| China | MN379944.1 | *Cygnus cygnus* |
| China | MK992450.1 | *Aves* |
| China | MK992455.1 | *Aves* |
| China | MK992412.1 | *Aves* |
